# New Aspects on *Listeria monocytogenes* ST5-ECVI Predominance in a Heavily Contaminated Cheese Processing Environment

**DOI:** 10.3389/fmicb.2018.00064

**Published:** 2018-02-01

**Authors:** Meryem Muhterem-Uyar, Luminita Ciolacu, Karl-Heinz Wagner, Martin Wagner, Stephan Schmitz-Esser, Beatrix Stessl

**Affiliations:** ^1^Department for Farm Animals and Veterinary Public Health, Institute of Milk Hygiene, Milk Technology and Food Science, University of Veterinary Medicine, Vienna, Austria; ^2^Department of Nutritional Sciences, Faculty of Life Sciences, University of Vienna, Vienna, Austria; ^3^Department of Animal Science, Iowa State University, Ames, IA, United States

**Keywords:** *Listeria monocytogenes*, food processing environment, persistence, multi locus sequence type, whole genome sequencing, plasmid

## Abstract

The eradication of *Listeria monocytogenes* from food chains is still a great challenge for the food industry and control authorities since some clonal complexes (CCs) are either better adapted to food processing environments (FPEs) or are globally widespread. In this work, we focus on the in-house evolution of *L. monocytogenes* genotypes collected from a heavily contaminated FPE whose contamination pattern underwent a massive and yet unexplained change. At the beginning of the sampling in 2010, a high variety of most likely transient *L. monocytogenes* genotypes was detected belonging to sequence type (ST) 1, ST7, ST21, ST37. After several efforts to intensify the hygiene measures, the variability was reduced to *L. monocytogenes* ST5 that was dominant in the following years 2011 and 2012. We aimed to elucidate possible genetic mechanisms responsible for the high abundance and persistence of ST5 strains in this FPE. Therefore, we compared the genomes of six *L. monocytogenes* ST5 strains to the less frequently occurring transient *L. monocytogenes* ST37 and ST204 from the same FPE as well as the highly abundant ST1 and ST21 isolated in 2010. Whole genome analysis indicated a high degree of conservation among ST5 strains [average nucleotide identity (ANI) 99.93–99.99%; tetranucleotide correlation 0.99998–0.99999]. Slight differences in pulsed field gel electrophoresis (PFGE) patterns of two ST5 isolates could be explained by genetic changes in the tRNA-Arg-TCT prophages. ST5 and ST204 strains harbored virtually identical 91 kbp plasmids related to plasmid group 2 (pLM80 and pLMUCDL175). Interestingly, highly abundant genotypes present in the FPE in 2010 did not harbor any plasmids. The ST5 plasmids harbored an efflux pump system (*bcrABC* cassette) and heavy metal resistance genes possibly providing a higher tolerance to disinfectants. The pLM80 prototype plasmids most likely provide important genetic determinants for a better survival of *L. monocytogenes* in the FPE. We reveal short-term evolution of *L. monocytogenes* strains within the same FPE over a 3 year period and our results suggest that plasmids are important for the persistence of ST5 strains in this FPE.

## Introduction

The zoonotic agent *Listeria monocytogenes* is capable to switch from an environment-associated to a pathogenic lifestyle and poses a high risk for immunocompromised persons, pregnant women, neonates and the elderly (Freitag et al., 2009; Allerberger and Wagner, 2010).

Cheeses, especially fresh-, soft and semi-hard varieties were often found to be vehicles in listeriosis outbreaks in the past and recently (Schoder et al., 2014; Buchanan et al., 2017). The epidemiological investigation of cheese-related outbreaks revealed substantial deficiencies in hygiene or in applied manufacturing protocols, a lack of consistent *L. monocytogenes* monitoring in food lots and food processing environments (FPEs), and the underestimation of *L. monocytogenes* growth potential during storage (Rückerl et al., 2014; Buchanan et al., 2017; Center of Disease Control and Prevention (CDC), 2017^1^).

The proximity to urban and agricultural environments increases the chance of *L. monocytogenes* introduction into food processing facilities due to their natural reservoirs and contamination cycles in soil, manure, decaying vegetation and water (Vivant et al., 2013; Linke et al., 2014).

As common colonizers of FPEs, distinct *L. monocytogenes* subtypes are able to survive in inefficiently cleaned niches of equipment and adapt to several stress factors such as low temperature, osmotic pressure, low pH and sublethal concentrations of biocides (Melo et al., 2015). A high *L. monocytogenes* prevalence in FPEs is often reported, and a constant risk of *L. monocytogenes* transmission to consumers remains a central challenge to the food industry (Almeida et al., 2013; Ferreira et al., 2014; Stessl et al., 2014; Malley et al., 2015; Muhterem-Uyar et al., 2015).

*Listeria monocytogenes* consists of four evolutionary lineages (I, II, III, and IV). Most listeriosis cases are associated with genetic lineage I (serotype 1/2b, 4b) and genetic lineage II (serotype 1/2a, 1/2c) strains (Orsi et al., 2011). Epidemic clones (ECs), genetic similar isolates involved in temporally and geographically unrelated large outbreaks, were defined by multi-virulence-locus sequence typing (MvLST) focusing on six to eight virulence associated genes (Chen et al., 2007). Multilocus sequence typing (MLST), a reference method for defining clonal complexes (CCs) based on seven housekeeping genes, was a different approach to identify genetically related isolates and highly abundant outbreak associated clones (Cantinelli et al., 2013). The most prevalent CCs correspond to important outbreak associated ECs: CC1 and ECI, CC2 and ECIV, CC5 and ECVI, CC6 and ECII, CC7 and ECVII, CC8 and ECV harboring full prerequisites of virulence (e.g., *prfA*, internalin A and B; listeriolysin O and *actA*). Listeriolysin S (*Listeria* pathogenicity island; LIPI-3) present in genetic lineage I strains [CC1-6, 59, 77, 224 and sequence type (ST) 54] and LIPI-4 (cellobiose-family PTS system) limited to CC4, contribute to neural and placental invasiveness (Cotter et al., 2008; Maury et al., 2016; Moura et al., 2016).

The progressive expansion of some genetic lineage II CCs (CC121, CC9, CC8, CC7, CC37, CC155, CC177, and CC204), indicate a special adaptation to the environment and to food matrices (Fox et al., 2016; Knudsen et al., 2017; Maury et al., 2016; Moura et al., 2016).

So-called persistent *L. monocytogenes* strains were reported to be better environmentally adapted due to the presence of resistance markers on plasmids (e.g., plasmid group1: plM33; group 2: plMST6; plM5578, plM80; pLM6179), the presence of prophages (tRNA, *comK*, phiLMST6), premature stop codons in *inlA*, stress-survival islet 1 (SSI-1; in ST3, 7, 8, 9, 155, and 204) or SSI-2 (CC121), enhanced biofilm formation, and/or tolerance to disinfectants as benzalkonium chloride (BC) (Elhanafi et al., 2010; Lomonaco et al., 2013; Müller et al., 2013; Melo et al., 2015; Liang et al., 2016; Martínez-Suárez et al., 2016; Xu et al., 2016; Buchanan et al., 2017; Hingston et al., 2017; Knudsen et al., 2017; Kremer et al., 2017).

The combination of classical molecular subtyping methods such as pulsed-field gel electrophoresis (PFGE) with whole-genome sequencing (WGS) empowers both, epidemiological outbreak investigations and in-depth analysis of genetic markers for persistence (Gilmour et al., 2010; Buchanan et al., 2017; Chen et al., 2017).

This study focused on a group of *L. monocytogenes* genetic lineage I isolates (ST5), which became highly prevalent in a cheese processing environment within a 3 year investigation. Subtyping of *L. monocytogenes* ST5 isolates using PFGE revealed three similar but slightly distinct *Asc*I PFGE patterns. The FPE harbored initially a high variety of *L. monocytogenes* genotypes on food contact (FCS) and non-food contact surfaces (NFCS), which decreased after improved hygiene measurements, so that mainly drains remained positive (Rückerl et al., 2014). Within this follow-up study we aimed to gain insight into genetic markers possibly responsible for the dominance of ST5 *L. monocytogenes* isolates from this FPE. Therefore, we compared persistent (ST5, *n* = 6) to sporadically isolated *L. monocytogenes* (ST1, ST21, ST37, and ST204; *n* = 5) from the same habitat by a comparative WGS approach. Furthermore, we determined potential resistances to antimicrobials, the epidemic clone and virulence type and performed cell culture experiments to estimate the *in vitro* virulence potential (ST1, ST5, ST21, ST37, ST204 vs. ST9) of these isolates.

## Materials and Methods

### *L. monocytogenes* Isolates and Processing Facility Characteristics

The *L. monocytogenes* occurrence in an Austrian cheese processing facility was investigated during 2010 to 2012. Raw material (vegetables, bacon) and intermediate products were occasionally contaminated. Initially, the PFGE analysis revealed a heterogeneous picture of up to 17 different PFGE profiles corresponding to nine STs. The heterogeneity of PFGE-profiles decreased resulting in one predominant profile (FCP7, ST5) (Rückerl et al., 2014).

**FIGURE 1:**
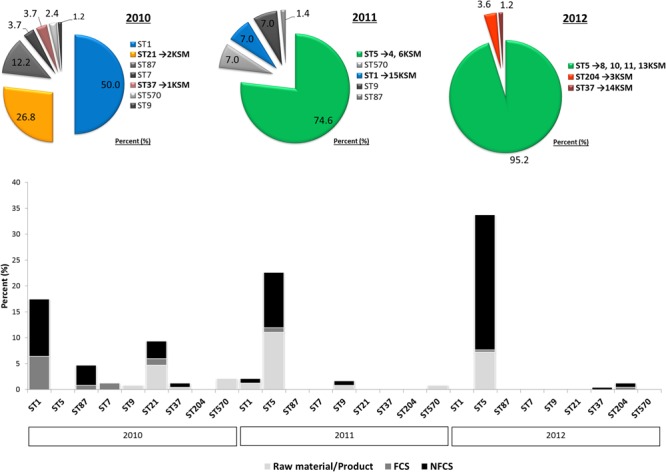
Decrease in *Listeria monocytogenes* genotypic heterogeneity in a cheese processing environment during 2010–2012 based on Rückerl et al. (2014). Different multi-locus sequence types are abbreviated by sequence type (ST). The strain are denoted by 1–15KSM. Further abbreviations: non-food contact surfaces (NFCS) and food-contact surfaces (FCS).

**FIGURE 2:**
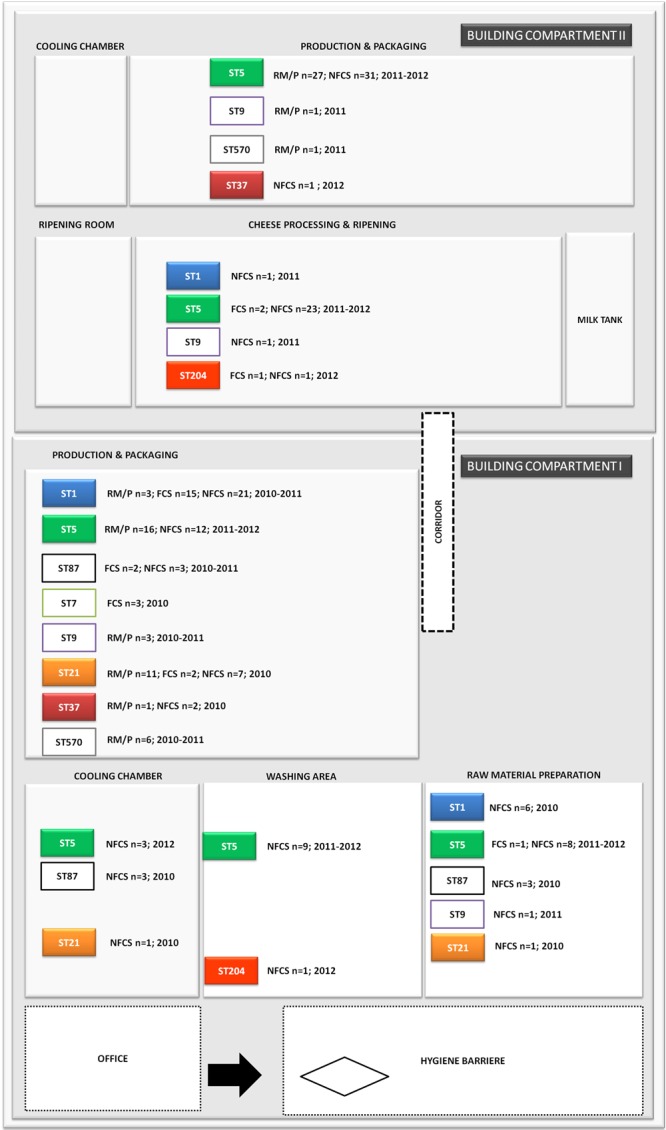
Distribution of *L. monocytogenes* sequence types (STs) in the building compartments I and II isolated from non-food contact surfaces (NFCS), food-contact surfaces (FCS) and raw materials (RM) or products (P) during the study phase (2010–2012).

During this study, a strain panel of 11 *L. monocytogenes* strains, isolated from the same FPE habitat (drains) but different building compartments within the FPE were included in the WGS comparison. The strain set comprised four representatives of the predominant *L. monocytogenes* PFGE-genotype FCP7 (ST5) isolated during 2011–2012 (strains 4, 6, 8, and 13KSM) and two slightly different subtypes, FCP7st1 (10KSM) and FCP7st2 (11KSM). Furthermore, two representatives of the PFGE-profile FCP8 (ST37) (1KSM and 14KSM), which were recurrently isolated and one representative of the initially most abundant subtypes in 2010: 15KSM (ST1) and 2KSM (ST21) were included. The ST1 and ST21 isolates represented 76.8% of all isolates in 2010. At the end of the investigative period, *L. monocytogenes* PFGE-profile FCP12 (ST204, 3KSM) was introduced in the FPE (**Figures 1, 2**) revealing ongoing contamination events in this FPE. The *L. monocytogenes* isolates were stored at the strain collection of the Institute for Milk Hygiene, Milk Technology and Food Science (University of Veterinary Medicine in Vienna).

### Multi-Locus Sequence Typing (MLST) and Multi-Virulence-Locus Sequence Typing (MVLST)

Multi-locus sequence typing of seven housekeeping loci (*abcZ, bglA, cat, dapE, dat, ldh*, and *lhkA*) was performed. ST were determined using the Institute Pasteur Database^2^. *L. monocytogenes* STs detected in this study were compared with *L. monocytogenes* MLST profiles stored in the Institute Pasteur isolate database^3^.

Multi-virulence-locus sequence typing was performed as described previously^4^ (Chen et al., 2007; Lomonaco et al., 2013) by amplification of intragenic regions of six virulence genes (*clpP, dal, inlB, inlC, lisR*, and *prfA*). Sequencing was performed by LGC Genomics (LGC, Berlin, Germany). Multiple sequence alignments were performed with Clustal Omega^5^ (Li et al., 2015) and compared with sequences from the *L. monocytogenes* MVLST database^4^.

### DNA Isolation, Whole Genome Sequencing and Genome Analysis

Genomic DNA from *L. monocytogenes* strains was isolated using NucleoSpin^®^ tissue kit (Macherey-Nagel, Düren, Germany) according to manufacturer’s instructions for Gram-positive bacteria. The genomes were sequenced by Illumina HiSeq2000 sequencing technology (Illumina Inc., San Diego, CA, United States) at the Campus Science Support Facilities (CSF) Next Generation Sequencing unit, Vienna, Austria using paired-end sequencing technology and 100 bp read length. For each strain, eight million reads were *de novo* assembled using the software SeqMan NGen^®^ (DNAStar, Madison, WI, United States). The average coverage for the strains was as follows: 295× (1KSM), 277× (2KSM), 359× (3KSM), 311× (4KSM), 294× (6KSM), 282× (8KSM), 296× (10KSM), 290× (11KSM), 289× (13KSM), 287× (14KSM) and 260× (15KSM). The final number of assembled contigs ranged from 23 to 44 and 1 to 5 for chromosomal DNA and plasmids, respectively (**Table 1**).

**Table 1:** Metadata of Listeria monocytogenes isolated from a cheese processing environment during 2010–2012.

Strain ID	Compartment^a^	Sampling area	Isolation date^b^	ST^c^	EC^d^	VT^e^	G+C content (%)	Genome assembly size (Mbp)	Contigs (Number)	Prophages (Number)	Plasmid (assembly size kbp)	SSI-1^f^	*LMOf2365_481^g^*	*bcrABC*^h^
1KSM	BI	Cooling chamber	(I) 2010	37		60	37.9	2.931	34	2	–	–	+	–
2KSM	BI	Raw material preparation	(III) 2010	21		123	37.9	2.911	26	1	–	–	+	–
15KSM	BI	Production and packaging	(III) 2010	1	I	20	37.9	2.915	41	1	–	–	+	–
4KSM	BI	Production and packaging	(I) 2011	5	VI	63	37.9	3.020	33	1	p4KSM (90.51)	+	–	+
6KSM	BII	Cheese processing and ripening	(III) 2011	5	VI	63	37.9	3.025	32	1	p6KSM (91.67)	+	–	+
8KSM	BI	Washing area	(I) 2012	5	VI	63	37.9	3.022	32	1	p8KSM (91.28)	+	–	+
3KSM	BI	Washing area	(I) 2012	204		10	37.9	3.033	23	2	p3KSM (92.04)	+	–	+
11KSM	BII	Cheese processing and ripening	(II) 2012	5	VI	63	37.9	3.024	44	1	p11KSM (90.28)	+	–	+
13KSM	BI	Washing area	(II) 2012	5	VI	63	37.9	3.024	28	1	p13KSM (91.55)	+	–	+
14KSM	BII	Production and packaging	(III) 2012	37		60	37.9	2.894	36	1	–	–	+	–
10KSM	BII	Production and packaging	(III) 2012	5	VI	63	37.9	3.043	41	1	p10KSM (91.03)	+	–	+

*^a^BI and BII, building compartment; ^b^Isolation date, quarterly periods are abbreviated with I, II, III, and IV; ^c^ST, sequence type; ^d^EC, epidemic clone; ^e^VT, Virulence type; ^f^SSI-1, stress survival islet 1, inserted between *lmo0443* and *lmo0449*, consisting of five genes (*lmo0444, lmo0445, pva, gadD1*, and *gadT1*); ^g^*LMOf2365_48*, uncharacterized SSI-1 homolog; ^h^*bcrABC*, plasmid encoded benzalkonium chloride resistance cassette.*

The chromosomal contigs were aligned to the following reference genomes using the “move contigs” option in MAUVE (Darling et al., 2010): EGD-e (GenBank accession number AL591824) for ST21, ST37 and ST204. SLCC2755 (GenBank accession number FR733646) for ST5 and F2365 (GenBank accession number AE017262I) for ST1. The plasmid contigs were ordered to the plasmid N1-011A (GenBank accession number CP006611). All chromosome and plasmid contigs were connected by using the spacer “nnnnnnn” to maintain the desired order during annotation. The annotation of aligned contigs was achieved with the fully automated service RAST^6^ (Aziz et al., 2008; Overbeek et al., 2014). Details about bacterial strains (*n* = 11) including basic assembly and annotation information are listed in **Table 1**.

### Comparative Genome Analysis

Multiple genome, prophage and plasmid alignments were performed by applying the progressive Mauve software tool (Darling et al., 2010). Average nucleotide identities between genomes and plasmids and correlation indexes of tetra-nucleotide signatures (Pairwise Tetra calculation) was determined using the JSpeciesWS Web Server (Richter et al., 2016). Comparison of present or absent genes among strains was determined by using the BLAST options of RAST^6^. Genome comparisons and predictions of homologous proteins were verified with BLASTN, BLASTP, uniProt and by applying pairwise sequence alignments based on BLAST^7^ (Altschul et al., 1990; Camacho et al., 2009). Similar to a previous study (Kuenne et al., 2013) we used a similarity cut-off of 60% amino acid identity and 80% coverage for identification of homologous proteins. Available *L. monocytogenes* genomes sequences in the NCBI database were compared to the genomes included in this study for their symmetric identity^8^.

### Accession Numbers

Genome sequences have been submitted to the NCBI and can be found under following GenBank accession numbers: JYOH00000000 (strain 1KSM), JYOI00000000 (strain 2KSM), JYNF00000000 (strain 3KSM), JYOJ00000000 (strain 4KSM), JYOL00000000 (strain 6KSM), JZBQ00000000 (strain 8KSM), JZHB00000000 (strain 10KSM), JZHC00000000 (strain 11KSM), JZCT00000000 (strain 13KSM), JYOS00000000 (strain 14KSM), JYOT00000000 (strain 15KSM).

### Antimicrobial Resistance Testing

The antimicrobial resistance (AMR) of *L. monocytogenes* isolated from the cheese FPE was tested by applying the commercially available Sensitre^TM^ Gram-positive plate assay (Thermo Fisher Scientific, Waltham, MA, United States). A panel of 18 antimicrobials at the concentrations indicated in parentheses included in the assay: erythromycin (ERY; 0.25 to 4 μg/ml), clindamycin (CLI; 0.12 to 2 μg/ml), quinupristin/dalfopristin (SYN; 0.12 to 4 μg/ml), daptomycin (DAP; 0.25 to 8 μg/ml), vancomycin (VAN; 1 to 128 μg/ml), tetracycline (TET; 2 to 16 μg/ml), ampicillin (AMP; 0.12 to 16 μg/ml), gentamicin (GEN; 2 to 16 μg/ml), rifampin (RIF; 0.5 to 4 μg/ml), levofloxacin (LEVO; 0.25 to 8 μg/ml), linezolid (LZD; 0.5 to 8 μg/ml), penicillin G (0.06 to 8 μg/ml), ciprofloxacin (CIP; 0.5 to 2 μg/ml), trimethoprim-sulfamethoxazole (SXT; 0.5/9.5 to 4/76 μg/ml), ceftriaxone (AXO; 8 to 64 μg/ml), gatifloxacin (GAT; 1 to 8 μg/ml), oxacillin+2% NaCl (OXA+, 0.25 to 8 μg/ml).

*Listeria monocytogenes* isolates (KSM1-KSM15) were grown on Mueller-Hinton agar (Oxoid) for 24 h at 37°C incubation. The overnight cultures were suspended in sterile saline solution (0.85% NaCl) to achieve a turbidity of a McFarland standard of 0.5 and then diluted 1:100 before use. The breakpoints for MICs and multi-drug resistance (MDR; resistance to two or more antibiotic classes) were determined according to EUCAST^9^ and Clinical and Laboratory Standards Institute (CLSI) standards.

### Virulence Tissue Culture Assays Using the Caco-2 Cell Line

The human intestinal adenocarcinoma cell line Caco-2 (ATCC^®^ HTB-37^TM^) was cultivated in Eagle’s minimum essential medium (MEM, Thermo Fisher Scientific, Waltham, MA, United States) supplemented with 10% fetal bovine serum, 2 mM/l L-glutamine, 1% (v/v) non-essential amino acids, and antibiotics (100 IU/ml penicillin, 100 mg/ml streptomycin, and 0.25 mg/ml amphotericin B) (all Sigma–Aldrich, St. Louis, MO, United States) at 37°C in a humidified atmosphere (95% relative humidity) containing 5% CO_2_.

One colony of *L*. *monocytogenes* [1, 2, 3, 4, 6, 15KSM and 5KSM] was inoculated in brain heart infusion complemented with yeast extract (BHI-Y, Merck) and cultivated for 8 h at 37°C. The bacterial culture was adjusted to OD_600_ 0.1 in 8 ml BHI-Y and grown for 32 h at 10°C without shaking mimicking natural contamination conditions in a cheese processing facility. Cell monolayers were infected with *L*. *monocytogenes* at a multiplicity of infection (MOI) of 25 for 1 h at 37°C. The cell monolayers were washed with Dulbecco’s Phosphate Buffered Saline (PBS; Thermo Fisher Scientific) and incubated in Eagle’s minimum essential medium (MEM), 10% FBS containing gentamicin (100 μg/ml) for 45 min (invasion) and 4 h (intracellular growth), respectively. The cells were lysed with 1 ml 0.1% Triton X-100 (Merck) and colony forming units (CFU) were determined by plating on tryptic soy agar (Biokar Diagnostics, Allonne, France) complemented with 0.6% yeast extract. The invasion efficiency (%) was calculated as mean CFU recovered after 45 min of gentamicin treatment divided by CFU of the inoculum. The intracellular growth coefficient (IGC) was calculated as follows: IGC = (intracellular bacteria_4h_-intracellular bacteria_45 min_)/intracellular bacteria_45 min_. Each experiment was performed in triplicate and repeated four times. Two reference strains EGDe (ST35) and QOC1 (ST403; Austrian Quargel outbreak clone 1) were included in the experimental setting. 5KSM (ST9) served as control strain with low invasion capacities due to a truncated *inlA* gene. A one way ANOVA (SPSS.20 software, SPSS Inc., Chicago, IL, United States) was undertaken to calculate the variance on the mean invasion and IGC of four independent experiments performed with each strain, and *post hoc* test (Tukey-HSD) was used to determine significant differences between the strains (*P* < 0.05).

## Results and Discussion

### Molecular Subtyping of *L. monocytogenes* Occurring in the Same FPE from 2010 to 2012

The investigation took place in a heavily contaminated FPE producing a variety of fresh and soft cheeses. *L. monocytogenes* was isolated from 19.5% of more than 1200 total swab and drain samples from both FCS and NFCS. The processing environment (drains, walls, doors, and floors) was found to be positive for *L. monocytogenes* during the whole investigation at a high rate (15.8%; Rückerl et al., 2014). These data demonstrate that the quality management procedures in the analyzed FPE were not efficient to reduce the contamination to an acceptable level. Due to the massive contamination and the high number of positive isolation events, this enterprise was particularly well suited to study the in-house *L. monocytogenes* population structure. MLST typing resulted in nine *L. monocytogenes* STs present to some extent during the study: ST37, ST1, ST5, ST7, ST87, ST21, ST570, ST9, and ST204 (**Figure 1**). For the genomic analysis, we chose representatives of ST1, ST5, ST21, ST37, and ST204 for the following reasons: The dominant clone ST5 was absent at the beginning of sampling in 2010, but increased massively in abundance in 2011 and 2012 when 74.6 and 95.2% of all tested isolates from all compartments were ST5 (**Figures 1, 2**). How this highly dynamic contamination could have happened remains unclear. An introduction of the ST5 strains from outside into the FPE during late 2010/early 2011 is highly likely. This occurrence and following dominance of ST5 strains implies that the sanitary advice was not followed completely. Chlor-free disinfectants including quaternary ammonium compounds and polyhexamethylene biguanide (polihexanide) were applied in excessive form and combined with floor carpets where disinfectants might have accumulated.

ST37 strains (indistinguishable by PFGE) were isolated at three occasions in the beginning and at a single occasion in 2012, always from the same compartment (production and packaging). The ST204 isolate (3KSM) was most likely introduced in 2012 and isolated at three occasions from two compartments (washing area and cheese processing and ripening; **Figure 2**). This ST became more abundant in 2013 (data not shown). ST1 (15KSM) and ST21 (2KSM) were highly abundant in the 1st year of sampling and represented 76.8% of all isolates from 2010 (**Figure 1**).

In comparison to MLST, PFGE-typing with *Asc*I revealed two subtypes among the six *L. monocytogenes* ST5 isolates, which were indistinguishable by PFGE and MLST: 4KSM, 6KSM, 8KSM, 10KSM, 13KSM; one band difference by PFGE but the same ST was found in 10KSM and 11KSM thus indicating some minor genetic changes in 10KSM and 11KSM-possibly in prophage regions (Gilmour et al., 2010; Rückerl et al., 2014).

Globally disseminated ST5 strains representing epidemic clone VI [virulence type (VT) 63] were isolated from geographically distant areas (e.g., Austria, Canada, Australia, Switzerland, Finland, China, and Chile), different origin (humans, animals, food, environment) and caused several United States multi-state listeriosis outbreaks (imitation crabmeat 1996; cantaloupe 2011; Hispanic style cheese and stone fruits 2014, ice cream 2015) (Schmid et al., 2014; Wang et al., 2015; Buchanan et al., 2017; Meier et al., 2017; Institute Pasteur MLST database^10^). Major differences among ST5/ECVI strains were observed with WGS/PFGE analysis (Chen et al., 2017) or *comK* prophage typing (Lomonaco et al., 2013) in the gain or loss of prophage regions such as tRNA-Arg TCT, *comK* and tRNA-Arg CCG prophages. Interestingly, *comK* prophages were absent in the cantaloupe outbreak strain 1 (Chen et al., 2017) and all ST5 strains included in this study. Zhang et al. (2016) reported a close genetic relatedness for ST5 strains from food and human cases in Shanghai, China circulating rather locally. Another *L. monocytogenes* genotype chosen for this study was ST37: ST37 strains were reported to undergo a recent clonal expansion and were predominantly isolated from Austrian soil, compost samples, plant material and water^10^ (Linke et al., 2014). ST37 strains included in this study were corresponding to VT61 in the MLVST database. Recent reports indicate sporadic isolation of ST37 in products of animal origin (Schoder et al., 2014; Rodríguez-Lázaro et al., 2015; Kvistholm Jensen et al., 2016) but also few clinical cases have been reported (Maury et al., 2016). ST204 (VT10) were up to now isolated from the cheese and meat production chain in the Czechia and Australia (Stessl et al., 2014; Fox et al., 2016), European fish FPE (MLST database Institute Pasteur) and Danish FPE (Knudsen et al., 2017). ST1/ECI is globally the most prevalent genotype and was recently involved in the United States caramel apple outbreak (2014–2015) (Chen et al., 2016; Maury et al., 2016). ST21 (VT123) is strongly associated to wild animals (hare, birds) and the environment (soil) (Linke et al., 2014; MLST database Institute Pasteur). A Minimum spanning tree (MST) analysis of *L. monocytogenes* lineages I and II strains of this study, in comparison with the Institute Pasteur strain collection based on identical allelic *abcz* types including VTs and ECs is depicted in Supplementary Figure S1. All ST5 strains were identified as ECVI (VT63) and ST1 as ECI (VT20).

### Comparative Genome Analysis of Transient and Persistent *L. monocytogenes* STs

**FIGURE 3:**
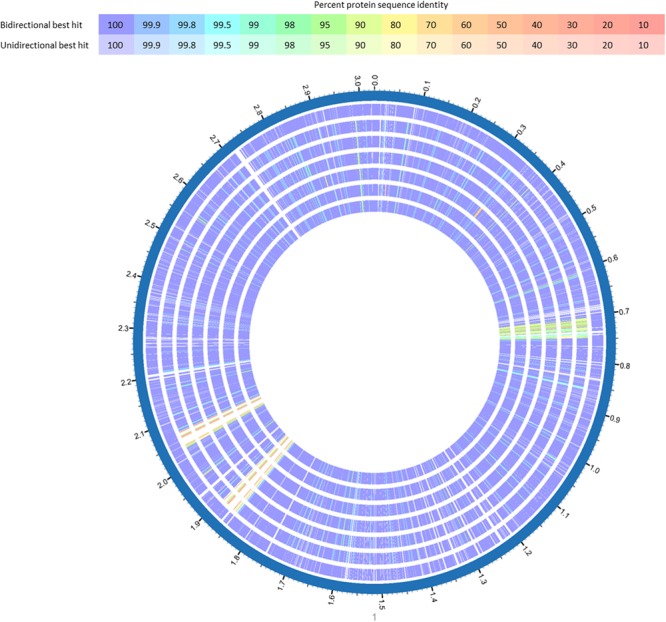
BlastP comparison of *L. monocytogenes* ST5 genomes from the same FPE compared with *L. monocytogenes* CFSAN029793. BlastP was performed in Patric (Wattam et al., 2017). The percent sequence identity is indicated in different colors for unidirectional and bidirectional BlastP hits. List of tracks, from outside to inside: *L. monocytogenes* CFSAN029793, *L. monocytogenes* 13KSM, *L. monocytogenes* 11KSM, *L. monocytogenes* 10KSM, *L. monocytogenes* 8KSM, *L. monocytogenes* 6KSM, *L. monocytogenes* 4KSM.

The WGS analysis revealed typical genomic features of *L. monocytogenes* such as assembly size – which do not represent the actual genome sizes because the genomes are not closed – ranging from 2.894 (14KSM) to 3.043 Mb (10KSM) and a genomic G+C content of 37.9% (den Bakker et al., 2010) (**Table 1**). Overall, all sequenced ST5 genomes are highly similar to each other (**Figure 3**). The average nucleotide identity (ANI) was >99.98% for 4KSM, 6KSM, 8KSM and 13KSM and >99.93% for 10KSM and 11KSM which had slightly different PFGE patterns (Supplementary Table S1). Also the Tetranucleotide analysis revealed highly similar genomes within the ST5 strains from this study, showing *r*^2^ values greater than 0.99998 (Supplementary Table S2), which is indicative of clonal relationships between the ST5 strains (Burall et al., 2016). The two ST37 genomes (1KSM, 14KSM) shared 99.94% ANI (Supplementary Figure S2). Schmid et al. (2014) reported a human listeriosis cluster caused by ST5 strains potentially linked to an Austrian fresh cheese facility or a meat product manufacturer (A and B). The authors identified in a core genome MLST phylogenetic approach for producer A and B a ≤19 and ≤8 gene difference from the human cluster but could not link the case directly due to missing quantitative data. We compared human and cheese isolates from producer A to our ST5 isolates and found also a high ANI (99.94%) but the tetranucleotide coefficient was 0.99992 indicating no clonal relationship (data not shown). A direct comparison with 1528 available genomes at the NCBI database revealed the highest symmetric identity for 4, 6, 8, and 13KSM (>99.7%). Interestingly, A45 isolated from food in Canada and LM05-00704 (unpublished genome Institute Pasteur; GenBank accession number: GCA001564595.1) were more similar (>99.4%) to 4, 6, 8, and 13KSM than 10 and 11KSM (99.34%) (Hingston et al., 2017). The comparison of 1KSM and 14KSM revealed a 98.5% symmetric identity^11^.

### Prophage Content and Conservation

The *L. monocytogenes* strains included in this study harbored one or two prophages, either located adjacent to tRNAs or inserted into the *comK* gene (3KSM, 15KSM) (**Table 2**). The highest homology (100%) between tRNA Arg-TCT prophages was found within ST5 strains (4, 6, 8, and 13KSM; approximately 43.69 kbp length). The ST5 PFGE subtype strains 10 and 11KSM both harbor larger and thus slightly distinct tRNA Arg-TCT prophage regions (approximately 61 and 47 kbp) (**Table 2** and Supplementary Table S3), which could be responsible for the slightly different PFGE patterns of 10KSM and 11KSM compared to the other ST5 strains. The isolate 14KSM (ST37) differed from 1KSM (ST37) by the absence of the tRNA-Ser-CGA prophage (**Table 2** and Supplementary Figure S2) suggesting that the tRNA Ser-CGA prophage was lost in 14KSM during the time of sampling. Rearrangements in prophage regions and hypervariable hotspots were identified as major drivers for rapid niche adaptation of outbreak related (CC8; ECV strains 08-5578 and 08-5923) or persistent *L. monocytogenes* in the meat chain (Gilmour et al., 2010; Verghese et al., 2011; Kuenne et al., 2013). Prophages are most often conserved among outbreak related isolates. Highly similar but unrelated genotypes can be differentiated by phage typing tools (e.g., PHAST, PHASTER, comK typing) and support the identification of listeriosis outbreaks (Lomonaco et al., 2013; Chen et al., 2017). Based on the differences in prophage content in some of the strains in this study, particularly the different tRNA Arg-TCT prophages in the ST5 PFGE subtypes 10 and 11KSM, it is tempting to speculate that these changes in prophages were detrimental for their adaptation and survival to this particular FPE; and thus subsequently these two subtypes got lost from the FPE. This is in line with recent reports describing an important role of prophages for adaptation and survival under stress conditions (Wang et al., 2010; Hingston et al., 2017) or an increased competitiveness (Burns et al., 2015). Recently, we (Schmitz-Esser et al., 2015) described a high similarity of persistent ST121 strains isolated from different FPEs in their prophage regions. The only differences in their genomes were caused by putative rearrangements of prophages (strain 6179) in hypervariable hotspot 7 (*lmo0458-lmo0480*). Similar to what we stated above, it is conceivable that these rearrangements were detrimental for the survival of 10KSM and 11KSM strains in this FPE, as these changes occurred only temporarily during 2011.

**Table 2:** Identified prophages in the L. monocytogenes genomes included in this study.

	1KSM	2KSM	15KSM	4KSM	6KSM	8KSM	3KSM	11KSM	13KSM	14KSM	10KSM
ST^a^	37	21	1	5	5	5	204	5	5	37	5
tRNA-Arg-TCT prophage	44.3^b^ (1)^c^			43.7 (1)	43.7 (1)	43.7 (1)	44 (2)	47.2 (6)	43.7 (1)	44 (2)	61.8 (10)
tRNA-Ser-CGA prophage	33.7 (1)										
tRNA-Arg-CCG prophage		39.9 (1)									
*comK* prophage			39.1 (1)				35.1 (3)				

*^a^ST, sequence type; ^b^Assembly size (kbp); ^c^Number of contigs.*

### Plasmids in ST5 and ST204

**FIGURE 4:**
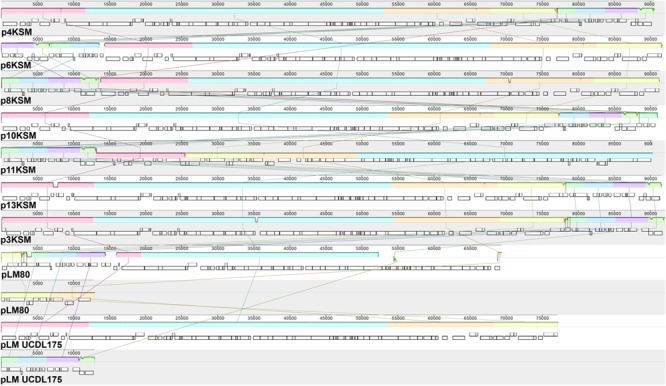
MAUVE alignment of *L. monocytogenes* 4, 6, 8, 10, 11, 13KSM (ST5), 3KSM (ST204) and reference plasmids plmUCDL175 (ST204, Fox et al., 2016) and pLM80 (ST6; reference strain H7858). Homologous regions are shown in the same color. The height of the similarity profile within each block corresponds to the average level of conservation (Darling et al., 2010). The two contigs of pLM80 and pLMUCDL175 are shown separately.

Putatives plasmids assigned to *Listeria* group 2 (Kuenne et al., 2010) were detected in all ST5 and ST204 strains with assembly sizes ranging from 90.2 to 92.0 kb (**Table 1**). Plasmids of ST5 and ST204 were virtually identical and showed more than 99.97% nucleotide identity to each other with more than 99.3% coverage (**Figure 4** and Supplementary Table S3). The ANI of ST5 and ST204 plasmids was 99.98% when compared to pLM80, a plasmid initially identified in the 1998–1999 multistate outbreak strain H7550 (ST6/ECII) involving contaminated hot dogs (Chen et al., 2016). High homologies (99.97%) were identified between pLM80-like plasmids isolated in this study and pUCDL_175, a plasmid harbored by a ST204 strain highly abundant in Australia (**Figure 4**) (Fox et al., 2016). Furthermore, a close relationship to N1-011A, a large uncharacterized plasmid (149 kbp) isolated from a ST3 strain, was identified (nucleotide identity >99%, coverage 68%). The high similarity of the ST5 and ST204 plasmids in strains from the FPE, suggests that the plasmids might have been transferred from the ST5 strains to the ST204 strains which occurred in the FPE in 2012 and became more abundant in 2013 (data not shown). Alternatively, the presence of almost identical plasmids (pLM80 prototypes) in different CC (5, 6, 9, 121, 204) and serotypes (1/2b, 4b) which are globally spread, suggests that a high selective pressure acts on these plasmids and they probably confer additional stress response capabilities to their host strains (Fox et al., 2016; Liang et al., 2016; Xu et al., 2016; Hingston et al., 2017; Meier et al., 2017). Our findings are in line with recent studies suggesting that the presence of almost identical plasmids in strains from different geographic sources and years suggests important contribution of plasmids to survival in food and FPEs (Schmitz-Esser et al., 2015; Fagerlund et al., 2016; Fox et al., 2016). The ST5 and ST204 plasmids harbor putative type III restriction modification systems, possibly involved in the protection against foreign DNA, genes predicted to be involved in oxidative stress response (peroxidase, NADH-oxidoreductase), a heavy metal resistance operon, cold and osmotic stress (*clpB, clpL*), a multidrug detoxification system including the *bcrABC* cassette efflux pump system, responsible for increased benzalkonium chloride tolerance (Elhanafi et al., 2010; Meier et al., 2017) which seem to have a positive impact on the niche adaptation and long-term persistence. During hygiene measures at this FPE, a huge variety of different sanitizers and disinfectants were applied. The testing for potential resistance to disinfectants showed an increased tolerance of the ST5 strains to BC and of the ST204 strain (3KSM) to Weiquat, a multi-component disinfectant including BC (Rückerl et al., 2014). The other strains from this FPE – which did not harbor the pLM80 related plasmid showed a lower tolerance toward disinfectants. This suggests a potential adaptation to the niche of isolation (drains) where disinfectant stress was high (Rückerl et al., 2014). Based on the presence of the *bcrABC* loci on the plasmids, it is highly likely that the plasmids contribute to the spread of ST5 and the occurrence of ST204 strains in this FPE. Of note, the most abundant *L. monocytogenes* STs found in this FPE in 2010: ST1 (15KSM) and ST21 (2KSM), and the sporadically isolated ST37 strains (1KSM, 14KSM) did not harbor plasmids, a *bcrABC* cassette, or other genes involved in tolerance to disinfectants (*Tn*6188, *qacH, emrB, emrE*).

### Presence of Virulence, Stress and Biofilm Associated Genes

The scanning of the genomes for 81 known virulence associated genes (reference genome EGDe; e.g., internalin genes, *prfA, plcA, hly, mpl, actA, plcB, uhpT*, and *bsh*) and biofilm markers revealed that they were to a majority present in all *L. monocytogenes* test strains (Supplementary Tables S4, S5). SSI-1 responsible for increased acid and salt adaptation also in human hosts, was present in both ST5 and ST204 strains. Lineage II strains 1 and 14KSM (ST37), 15KSM (ST1), and 2KSM (ST21) contained the yet uncharacterized *lmof2365_0481* gene instead of SSI-1. Malekmohammadi et al. (2017) reported the clonality of SSI-1 in CC3, 5, 7, and 9 suggesting a higher tolerance toward salt and nisin.

### AMR-Results

Antimicrobial resistance testing of the *L. monocytogenes* strains (1–15KSM) showed susceptibility to a broad range of antibiotic classes. Especially for VAN (glycopeptides antibiotic), TET (tetracycline), SXT (sulfonamide), RIF (rifamycine), GAT (4th generation fluoroquinolone), STR (aminoglycoside), and ERY (macrolide) no antibiotic resistance was observed (MIC below the test concentrations). All strains were resistant to a cyclic lipopeptide (DAP; MIC > 8 μg/ml). For some antibiotic substances MIC or MIC ranges were observed within the test concentrations and are provided in **Table 3**.

**Table 3:** Minimum inhibitory concentration (MIC) or MIC range (μg/ml) of the antimicrobial agents against *L. monocytogenes* test strains.

Antibiotic	Antibiotic class		MIC or MIC range (μg/ml)					
		Concentration (μg/ml)	ST1 (15KSM)	ST5 (4–13KSM)	ST9 (5KSM)	ST21 (2KSM)	ST37 (1, 14KSM)	ST204 (3KSM)
Penicillin (PEN)	β-lactam antibiotic	0.06-8	0.5	0.25	0.12	0.12		
Ampicillin (AMP)	β-lactam antibiotic	0.12-16	0.25					
Oxacillin (OXA+)	β-lactam antibiotic	0.25-8	4	2	1	1	0.25–1	0.5
Ceftriaxone (AXO)	Cephalosporine (third generation)	8–64	>64	32	16			
Ciprofloxacin (CIP)	Fluoroquinolone (second generation)	0.5-2	2	1	1	1	0.5–1	1
Levofloxacin (LEVO)	Fluoroquinolone (third generation)	0.25-8	1	1	1	0.5	1	1
Clindamycin (CLI)	Lincosamide	0.12-2	1	1	1		0.12–0.5	1
Gentamicin (GEN)	Aminoglycoside	2–16	4					
Linezolid (LZD)	Oxazolidinone	0.5-8	4	1	1	1	1	1
Quinupristin/dalfopristin (SYN)	Streptogramin	0.12-4	0.5	1	0.5	0.25	0.25–0.5	0.5
Daptomycin (DAP)	Cyclic lipopeptides	0–25–8	>8	>8	>8	>8	>8	>8

Generally, ampicillin in combination with gentamicin and trimethoprim/sulfonamide as important antibiotics in the treatment of listeriosis were effective in all strains according to EUCAST and CLSI clinical breakpoints.

Multi-drug resistance resistance was detected in 15KSM (ST1, ECI) for cephalosporine (AXO > 64 μg/ml), fluoroquinolone (CIP > 2 μg/ml) and cyclic lipopeptide (DAP > 8 μg/ml). 15KMS indicated also a higher tolerance to OXA+ (β-lactam antibiotic; 16-fold increase), GEN (gentamicin; 2-fold increase) and LZD (linezolid; 4-fold-increase) in comparison to the other test strains. 15KSM associated to the globally most abundant CC1 showed increased MICs in several antibiotic substances which were not plasmid mediated.

ST 5 strains showed higher MICs for following antibiotics in comparison to genetic lineage II strains: OXA+ (β-lactam antibiotic; up to 8-fold increase), SYN (streptogramine; up to 4-fold increase), PEN (β-lactam antibiotic; 2-fold increase) and AXO (cephalosporine; 2-fold increase) (**Table 3**).

Natural resistance of *L. monocytogenes* to oxacillin and resistance to ciprofloxacin, linezolid, clindamycin, ampicillin and rifampicin were more often reported in genetic lineage I strains. The induction of AMR through sublethal stress exposure by low concentrated antimicrobials and heavy metals is very likely. Co-selection for benzalkoniumchloride, hydrogen peroxide, heavy metals and antibiotics (macrolides, cefotaxime, fluoroquinolone) is a cross resistance phenomenon in *L. monocytogenes* and is mediated by efflux pumps (*mdrL, lde, emrE*) (Conficoni et al., 2016; Martínez-Suárez et al., 2016; Komora et al., 2017). In our study the higher tolerance to antibiotics observed in genetic lineage I strains seemed not to be plasmid mediated. A further explanation of higher antibiotic tolerance in genetic lineage I strains could be the biphasic population response of cells including persister cell subpopulations (Buchanan et al., 2017). More research is needed to fully understand the natural and acquired antibiotic resistance in *L. monocytogenes* applying harmonized protocols to complement missing epidemiological cut-offs.

### Invasion Efficiency and Intracellular Growth in Caco-2 Cells

**FIGURE 5:**
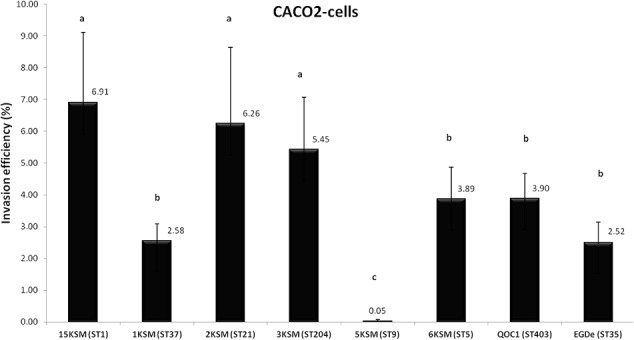
Invasion efficiency of *L. monocytogenes* strains included in this study in human Caco2 cells. Mean values and standard deviations of the four independent biological replicates are presented. Different letters indicate significant differences (*P* < 0.05) between the invasion efficiency of the strains.

**FIGURE 6:**
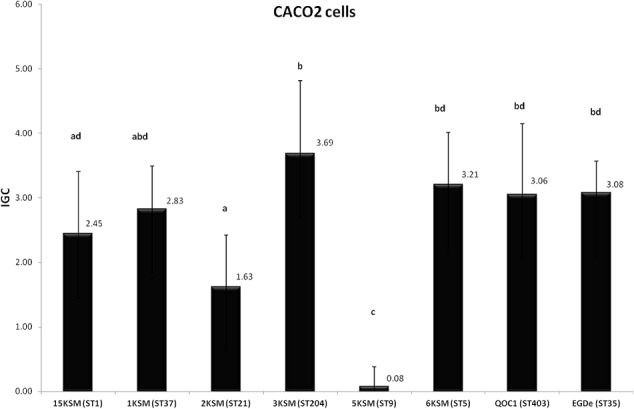
Intracellular growth of *L. monocytogenes* strains included in this study in human Caco2 cells. Mean values and standard deviations of the four independent biological replicates are presented. Different letters indicate significant differences (*P* < 0.05) between the invasion efficiency of the strains.

In the *in vitro* cell culture experiment six *L. monocytogenes* strains from the cheese FPE [1, 2, 3, 6, 15KSM and 5KSM (ST9, truncated *inlA*)] were compared to the reference strains EGDe (ST35) and the outbreak isolate QOC1 (ST403). Invasion efficiency was significantly higher (*P* < 0.05) for ST1, ST21 and ST204 (5.45–6.91%; group a) compared to ST5, ST37 and the reference strains EGDe and QOC1 (2.52–3.90%; group b) (**Figure 5**). The intracellular growth potential between the strains was also significantly different (*P* < 0.05): 3KSM (ST204) showed the highest intracellular growth comparable to 6KSM and the reference strains EGDe and QOC1 (group bd; **Figure 6**). 2KSM (ST21) had the lowest intracellular growth potential in comparison to the other test strains. These data suggest that all tested strains except 5KSM are able to replicate intracellularly once inside the host cell. Ciolacu et al. (2015) compared several STs (ST2, ST8, ST9, ST20, ST121, and ST155) isolated from illegally sold food products in Romania. The invasion efficiency and intracellular growth was comparable low for the ST9 isolates harboring a premature stop codon (PMSC) in the *inlA* gene. The other STs showed a high invasion and proliferation variability in Caco 2-cells comparable to our findings. The isolates included in this study harbored the full prerequisites for virulence and host adaptation, especially ST1 with the additional presence of listeriolysin S (Cotter et al., 2008). For risk assessment purposes it is relevant to estimate the virulence potential of *L. monocytogenes* contaminants. Strains harboring truncated *inlA* genes are assumed to have less potential to invade human epithelial cells (InlA/E-cadherin pathway) and would need a higher infective dose (3 log more cells to cause an infection) and might particularly affect mainly immunodeficient hosts with comorbidities (Maury et al., 2016; Buchanan et al., 2017).

## Conclusion

This study compared the genomes of transient and persistent *L. monocytogenes* strains isolated from the same cheese processing plant during a 3-year time period from 2010 to 2013. In line with previous results (den Bakker et al., 2010; Kuenne et al., 2013), the chromosomal backbone of the predominant ST5 strains isolated during 2011–2012 was highly conserved. Two ST5 subtypes (10 and 11KSM), each of them isolated only once, harbored slightly different tRNA-Arg-TCT prophages, suggesting that the changes in the prophage regions were detrimental for their survival in this FPE. Our data suggest that plasmids harboring – among others – a *bcrABC* resistance cassette against disinfectants were key for the adaptation and survival of the persistent ST5 strains in this FPE in spite of various concomitant hygiene measures in action in this FPE. A striking result was that *L. monocytogenes* ST5 (genetic lineage I; 1/2b, 3b) and ST204 strains (genetic lineage II; 1/2a, 3a) shared the same plasmids and additionally possessed SSI-1 for acid and salt adaptation. The occurrence of virtually identical plasmids in *L. monocytogenes* strains from various years and environments described here and in other recent studies strongly suggests the importance of plasmids for the survival of *L. monocytogenes* strains in various FPE (Fox et al., 2016; Liang et al., 2016; Xu et al., 2016; Hingston et al., 2017; Meier et al., 2017). More research will be needed in the future to determine the contribution of these plasmids to survival in FPE.

## Author Contributions

BS, SS-E, and MW conceived and designed the experiments. BS, MM-U, and LC performed all wet laboratory experiments. MM-U, SS-E, and BS conducted bioinformatics analyses. MM-U, SS-E, MW, and BS drafted the manuscript. K-HW reviewed the manuscript. All authors read and approved final the manuscript.

## Conflict of Interest Statement

The authors declare that the research was conducted in the absence of any commercial or financial relationships that could be construed as a potential conflict of interest.

## Footnotes

1 http://outbreakdatabase.com/search/?organism=Listeria+monocytogenes

2 http://bigsdb.pasteur.fr/listeria/listeria.html

3 http://bigsdb.pasteur.fr/perl/bigsdb/bigsdb.pl?db=pubmlst_listeria_isolates_public

4 https://sites.google.com/site/mvlstdatabase/home

5 http://www.ebi.ac.uk/Tools/msa/clustalo/

6 http://rast.nmpdr.org/

7 http://www.uniprot.org/

8 https://www.ncbi.nlm.nih.gov/genome/genomes/159?

9 http://www.eucast.org/clinical_breakpoints/

10 http://bigsdb.pasteur.fr/listeria/listeria.html

11 https://www.ncbi.nlm.nih.gov/genome/
