# Supplementary material for: New Aspects on Listeria monocytogenes ST5-ECVI Predominance in a Heavily Contaminated Cheese Processing Environment
Source: Front Microbiol. 2018 Feb 1;9:64. doi: 10.3389/fmicb.2018.00064 (PMC5810274; doi:10.3389/fmicb.2018.00064)
Supplement: Supplementary file 1 [file Data_Sheet_1.DOCX]

# Figure S1


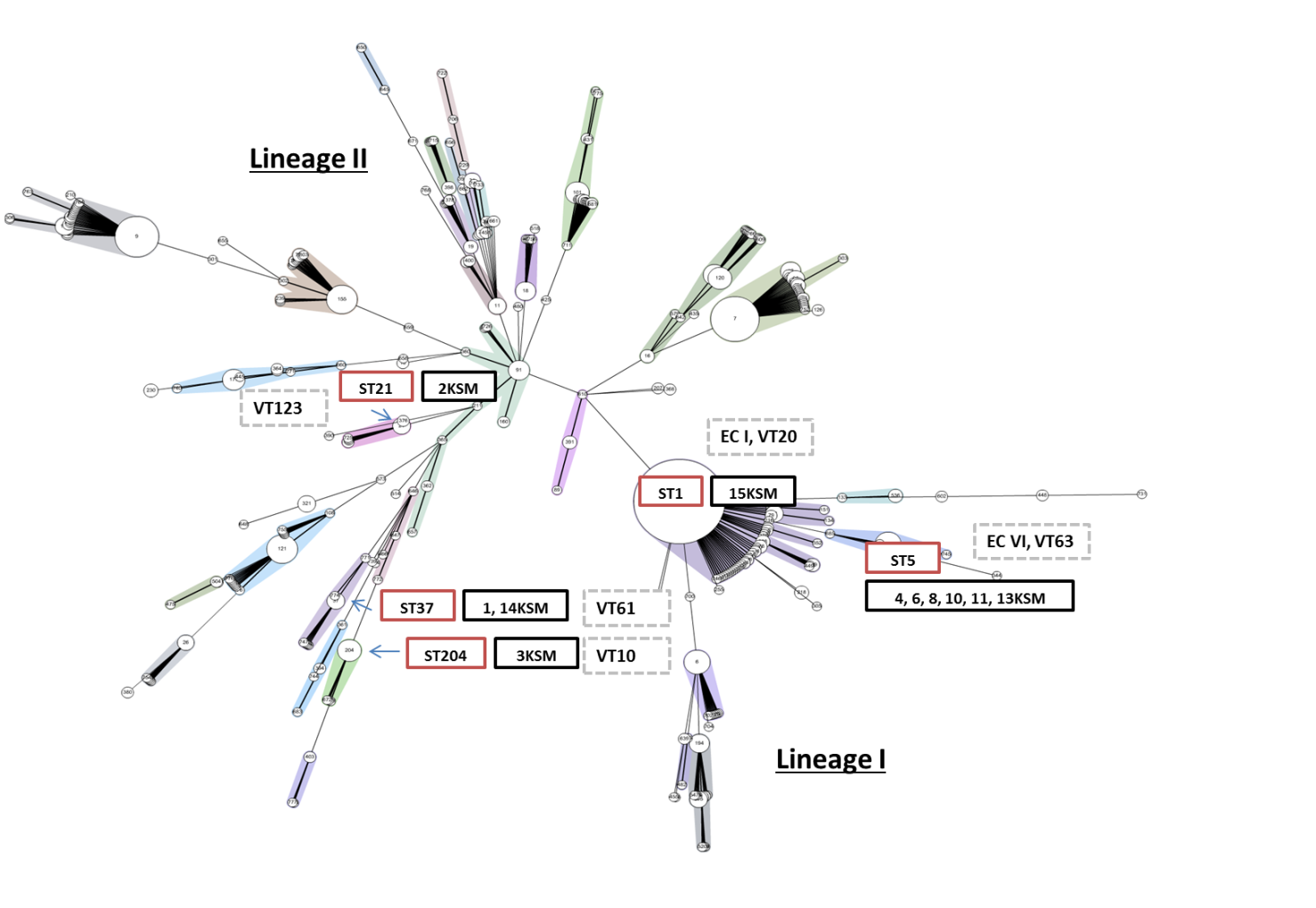


Multi-locus sequence typing (MLST) and multi-virulence-locus sequence typing (MVLST) of *L. monocytogenes* genetic lineage I (4, 6, 8, 10, 11, 13KSM) and II (1, 3 and 14KSM) isolated from a cheese processing environment. The sequence types were clustered according to the *abcZ* housekeeping genes using a minimum spanning tree (MST) tool available from the Institute Pasteur MLST database. Numbers within red and black boxes denote the corresponding ST and isolate number, respectively. *L. monocytogenes* strains were grouped into clonal complexes (CC; Clonal complexes are colored with a random color), defined as groups of profiles differing by no more than one gene from at least one other profile of the group (Ragon et al., 2008). Grey boxes indicate corresponding epidemic clones (EC) and virulence types (VT) (Lomonaco et al., 2013; https://sites.google.com/site/mvlstdatabase/home).

**Figure S2**

**
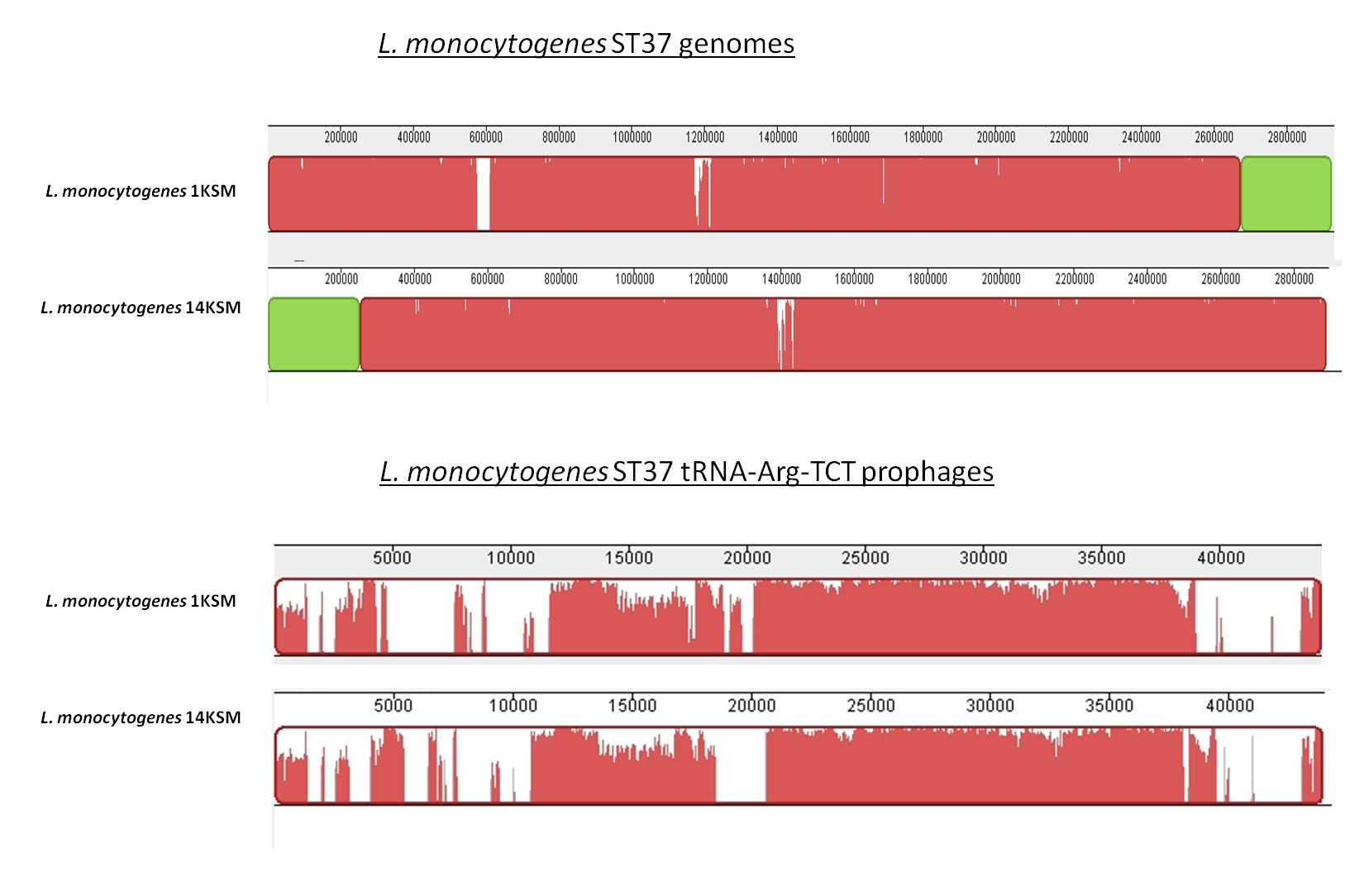
**

Mauve alignment of *L. monocytogenes* ST37 genomes (1 and 14KSM) and corresponding tRNA-Arg-TCT prophages (Darling et al., 2010).

**Figure S3**

**
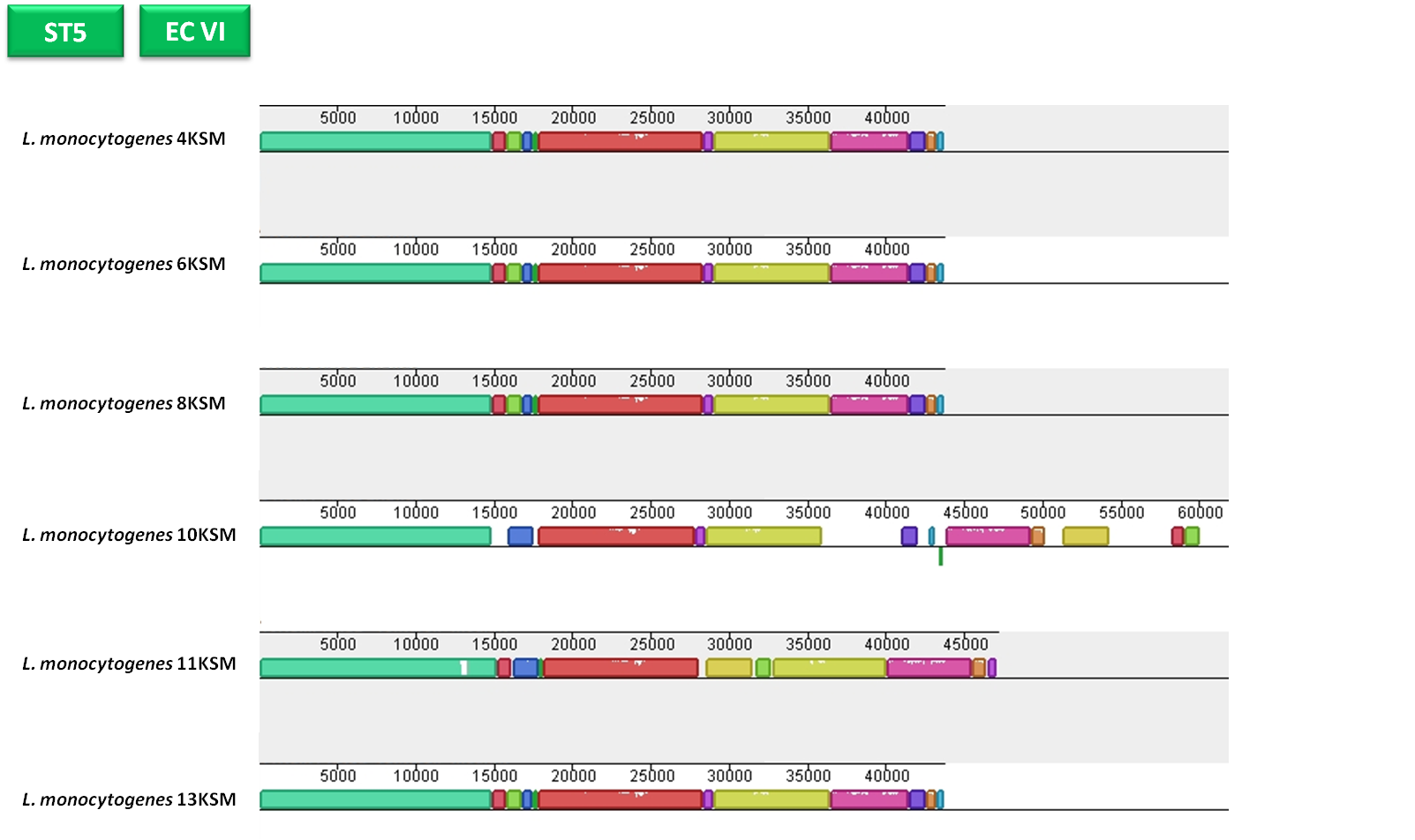
**

MAUVE alignment of *L. monocytogenes* ST5 tRNA-Arg-TCT prophages. Homologous regions have the same color and the height of the blocks correlate with the conservation level of the regions for each prophage. ST5 tRNA-Arg-TCT prohages were identical with the exception of 10 and 11KSM (subtypes in PFGE analysis). 10 and 11KSM prophages were assembled into several contigs and therefore aligned to the prophage of 4KSM to find a theoretical contig order (Darling et al., 2010).

**Table S1. Average nucleotide identity (ANI) of ST5 *L. monocytogenes* genomes from the same FPE.**

|  | **4KSM** | **6KSM** | **8KSM** | **10KSM** | **11KSM** | **13KSM** |
| --- | --- | --- | --- | --- | --- | --- |
| **4KSM** |  | **99.98** (99.49) | **99.98** (99.61) | **99.96** (99.61) | **99.93** (99.42) | **99.99** (99.60) |
| **6KSM** | **99.98** (99.72) |  | **99.99** (99.80) | **99.95** (99.79) | **99.94** (99.56) | **99.99** (99.77) |
| **8KSM** | **99.98** (99.94) | **99.99** (99.86) |  | **99.96** (99.99) | **99.94** (99.78) | **99.99** (99.97) |
| **10KSM** | **99.96** (99.70) | **99.96** (99.62) | **99.96** (99.75) |  | **99.96** (99.75) | **99.96** (99.74) |
| **11KSM** | **99.93** (99.76) | **99.94** (99.65) | **99.94** (99.79) | **99.96** (99.99) |  | **99.94** (99.78) |
| **13KSM** | **99.99** (99.94) | **99.99** (99.86) | **99.99** (99.99) | **99.96** (99.98) | **99.94** (99.79) |  |

The ANI values calculated by using the MUMmer software in the JSpeciesWS web server are shown in percent. The percentage of genomes covered is shown in brackets.

**Table S2. Tetranucleotide Analysis of ST5 *L. monocytogenes* genomes from the same FPE.**

|  | **4KSM** | **6KSM** | **8KSM** | **10KMS** | **11KMS** | **13KSM** |
| --- | --- | --- | --- | --- | --- | --- |
| **4KSM** |  | 0.99998 | 0.99999 | 0.99998 | 0.99998 | 0.99998 |
| **6KSM** | 0.99998 |  | 0.99998 | 0.99998 | 0.99998 | 0.99998 |
| **8KSM** | 0.99999 | 0.99998 |  | 0.99998 | 0.99999 | 0.99999 |
| **10KMS** | 0.99998 | 0.99998 | 0.99998 |  | 0.99998 | 0.99998 |
| **11KMS** | 0.99998 | 0.99998 | 0.99999 | 0.99998 |  | 0.99998 |
| **13KSM** | 0.99998 | 0.99998 | 0.99999 | 0.99998 | 0.99998 |  |

R^2^ values of Tetranucleotide Analysis done with the JSpeciesWS webserver are shown.

**Table S3. Average nucleotide identity (ANI) of ST5 and related *L. monocytogenes* plasmids.**

|  | **p4KSM** | **p6KSM** | **p8KSM** | **p10KSM** | **p11KSM** | **p13KSM** | **p3KSM** | **pUCDL_175** | **pLM80** |
| --- | --- | --- | --- | --- | --- | --- | --- | --- | --- |
| **MLST Sequence Type** | ST5 | ST5 | ST5 | ST5 | ST5 | ST5 | ST204 | ST204 | ST6 |
| **p4KSM** |  | **100.00** (99.98) | **100.00** (99.98) | **99.99** (99.98) | **100.00** (99.26) | **99.98** (99.98) | **99.98** (99.40) | **99.99** (99.34) | **99.98** (89.79) |
| **p6KSM** | **100.00** (99.39) |  | **100.00** (99.97) | **99.99** (99.97) | **100.00** (99.25) | **99.98** (99.97) | **99.98** (99.98) | **99.98** (99.45) | **99.98** (89.91) |
| **p8KSM** | **100.00** (99.40) | **100.00** (99.98) |  | **99.99** (99.98) | **100.00** (99.26) | **99.98** (99.98) | **99.99** (99.98) | **99.98** (99.36) | **99.98** (89.88) |
| **p10KSM** | **99.99** (99.40) | **99.99** (99.98) | **99.99** (99.98) |  | **99.99** (99.25) | **99.98** (99.98) | **99.98** (99.98) | **99.98** (99.42) | **99.98** (89.84) |
| **p11KSM** | **100.00** (99.39) | **100.00** (99.98) | **100.00** (99.98) | **99.99** (99.98) |  | **99.98** (99.98) | **99.98** (99.26) | **99.99** (99.45) | **99.98** (89.76) |
| **p13KSM** | **99.98** (99.40) | **99.98** (99.98) | **99.98** (99.98) | **99.98** (99.98) | **99.98** (99.26) |  | **99.97** (99.98) | **99.97** (99.47) | **99.98** (89.90) |
| **p3KSM** | **99.98** (99.40) | **99.98** (99.98) | **99.99** (99.98) | **99.98** (99.98) | **99.98** (99.26) | **99.97** (99.98) |  | **99.98** (99.98) | **99.99** (89.96) |
| **pUCDL_175** | **99.99** (99.41) | **99.98** (100.00) | **99.98** (100.00) | **99.98** (100.00) | **99.99** (99.27) | **99.97** (100.00) | **99.98** (99.98) |  | **99.95** (89.76) |
| **pLM80** | **99.98** (98.65) | **99.98** (99.29) | **99.98** (99.29) | **99.98** (99.29) | **99.98** (98.49) | **99.98** (99.29) | **99.99** (89.96) | **99.95** (98.92) |  |

The ANI values calculated by using the MUMmer software in the JSpeciesWS web server are shown in percent. The percentage of plasmids covered is shown in brackets.

**Table S4. Presence and absence of 81 virulence-associated genes in *L. monocytogenes* strains investigated in this study.**

|  |  | ST5 | | | | | | ST1 | ST37 | | ST204 |
| --- | --- | --- | --- | --- | --- | --- | --- | --- | --- | --- | --- |
| **Gene designation** | **EGD-e homologue** | **4KSM** | **6KSM** | **8KSM** | **10KSM** | **11KSM** | **13KSM** | **15KSM** | **1KSM** | **14KSM** | **3KSM** |
| *agrA* | *lmo0051* | +^a^ | + | + | + | + | + | + | + | + | + |
| *ctaP* | *lmo0135* | + | + | + | + | + | + | + | + | + | + |
| *prfA* | *lmo0200* | + | + | + | + | + | + | + | + | + | + |
| *plcA* | *lmo0201* | + | + | + | + | + | + | + | + | + | + |
| *hly* | *lmo0202* | + | + | + | + | + | + | + | + | + | + |
| *mpl* | *lmo0203* | + | + | + | + | + | + | + | + | + | + |
| *actA* | *lmo0204* | + | + | + | + | + | + | + | + | + | + |
| *plcB* | *lmo0205* | + | + | + | + | + | + | + | + | + | + |
| *lmo0206* | *lmo0206* | + | + | + | + | + | + | + | + | + | + |
| *ctsR* | *lmo0229* | + | + | + | + | + | + | + | + | + | + |
| *lmo0257* | *lmo0257* | + | + | + | + | + | + | + | + | + | + |
| *inlH/inlC2* | *lmo0263* | + | + | + | + | + | + | + | + | + | + |
| *htrA* | *lmo0292* | + | + | + | + | + | + | + | + | + | + |
| *vip* | *lmo0320* | + | + | + | + | + | + | + | - | - | - |
| *pgdA* | *lmo0415* | + | + | + | + | + | + | + | + | + | + |
| *inlA* | *lmo0433* | + | + | + | + | + | + | + | + | + | + |
| *inlB* | *lmo0434* | + | + | + | + | + | + | + | + | + | + |
| *lntA* | *lmo0438* | + | + | + | + | + | + | + | + | + | + |
| *lmo0540* | *lmo0540* | + | - | + | + | + | + | + | + | + | + |
| *iap* | *lmo0582* | + | + | + | + | + | + | + | + | + | + |
| *secA2* | *lmo0583* | + | + | + | + | + | + | + | + | + | + |
| *lmo0604* | *lmo0604* | + | + | + | + | + | + | + | + | + | + |
| *mogR* | *lmo0674* | + | + | + | + | + | + | + | + | + | + |
| *lmo0788* | *lmo0788* | + | + | + | + | + | + | + | + | + | + |
| *uhpT* | *lmo0838* | + | + | + | + | + | + | + | + | + | + |
| *sigB* | *lmo0895* | + | + | + | + | + | + | + | + | + | + |
| *lmo0915* | *lmo0915* | + | + | + | + | + | + | + | + | + | + |
| *lplA1* | *lmo0931* | + | + | + | + | + | + | + | + | + | + |
| *fri* | *lmo0943* | + | + | + | + | + | + | + | + | + | + |
| *dltA* | *lmo0974* | + | + | + | + | + | + | + | + | + | + |
| *auto* | *lmo1076* | + | + | + | + | + | + | - | + | + | + |
| *lmo1081* | *lmo1081* | + | + | + | + | + | + | - | + | + | + |
| *lmo1082* | *lmo1082* | + | + | + | + | + | + | - | + | + | + |
| *lmo1099* | *lmo1099* | -^b^ | - | - | - | - | - | - | - | - | - |
| *lmo1102* | *lmo1102* | - | - | - | - | - | - | - | - | - | - |
| *tig* | *lmo1267* | + | + | + | + | + | + | + | + | + | + |
| *sipX* | *lmo1269* | + | + | + | + | + | + | + | + | + | + |
| *sipZ* | *lmo1271* | + | + | + | + | + | + | + | + | + | + |
| *inlK* | *lmo1290* | + | + | + | + | + | + | + | + | + | + |
| *oat* | *lmo1291* | + | + | + | + | + | + | + | + | + | + |
| *hfq* | *lmo1295* | + | + | + | + | + | + | + | + | + | + |
| *tcsA* | *lmo1388* | + | + | + | + | + | + | + | + | + | + |
| *bilE* | *lmo1421* | + | + | + | + | + | + | + | + | + | + |
| *lmo1438* | *lmo1438* | + | + | + | + | + | + | + | + | + | + |
| *sod* | *lmo1439* | + | + | + | + | + | + | + | + | + | + |
| *lmo1521* | *lmo1521* | + | + | + | + | + | + | + | + | + | + |
| *relA* | *lmo1523* | + | + | + | + | + | + | + | + | + | + |
| *lmo1601* | *lmo1601* | + | + | + | + | + | + | + | + | + | + |
| *lmo1602* | *lmo1602* | + | + | + | + | + | + | + | + | + | + |
| *perR* | *lmo1683* | + | + | + | + | + | + | + | + | + | + |
| *mprF* | *lmo1695* | + | + | + | + | + | + | + | + | + | + |
| *adeC* | *lmo1742* | + | + | + | + | + | + | + | + | + | + |
| *virR* | *lmo1745* | + | + | + | + | + | + | + | + | + | + |
| *inlC* | *lmo1786* | + | + | + | + | + | + | + | + | + | + |
| *stp* | *lmo1821* | + | + | + | + | + | + | + | + | + | + |
| *fbpA* | *lmo1829* | + | + | + | + | + | + | + | + | + | + |
| *lmo1855* | *lmo1855* | + | + | + | + | + | + | + | + | + | + |
| *fur* | *lmo1956* | + | + | + | + | + | + | + | + | + | + |
| *lmo2026* | *lmo2026* | + | + | + | + | + | + | + | + | + | + |
| *lmo2048* | *lmo2048* | + | + | + | + | + | + | + | + | + | + |
| *bsh* | *lmo2067* | + | + | + | + | + | + | + | + | + | + |
| *lmo2114* | *lmo2114* | + | + | + | + | + | + | + | + | + | + |
| *lmo2115* | *lmo2115* | + | + | + | + | + | + | + | + | + | + |
| *dacA* | *lmo2120* | + | + | + | + | + | + | + | + | + | + |
| *lmo2157* | *lmo2157* | + | + | + | + | + | + | + | + | + | + |
| *lmo2177* | *lmo2177* | + | + | + | + | + | + | + | + | + | + |
| *svpA* | *lmo2185* | + | + | + | + | + | + | + | + | + | + |
| *oppA* | *lmo2196* | + | + | + | + | + | + | + | + | + | + |
| *fabF* | *lmo2201* | + | + | + | + | + | + | + | + | + | + |
| *lmo2203* | *lmo2203* | + | + | + | + | + | + | + | + | + | + |
| *clpB* | *lmo2206* | + | + | + | + | + | + | - | + | + | + |
| *prsA2* | *lmo2219* | + | + | + | + | + | + | + | + | + | + |
| *lmo2439* | *lmo2439* | + | + | + | + | + | + | + | + | + | + |
| *gap* | *lmo2459* | + | + | + | + | + | + | + | + | + | + |
| *degU* | *lmo2515* | + | + | + | + | + | + | + | + | + | + |
| *lmo2522* | *lmo2522* | + | + | + | + | + | + | + | + | + | + |
| *ami* | *lmo2558* | + | + | + | + | + | + | + | **-** | **-** | + |
| *murA* | *lmo2691* | + | + | + | + | + | + | + | + | + | + |
| *lmo2713* | *lmo2713* | + | + | + | + | + | + | + | + | + | + |
| *lmo2714* | *lmo2714* | + | + | + | + | + | + | + | + | + | + |
| *inlJ* | *lmo2821* | + | + | + | + | + | + | + | + | + | + |

^a^+, present gene/^b^-, absent gene; cutoff of 60% amino acid identity for present genes according to Kuenne et al., 2013.

**Supplement Table 5. Presence and absence of biofilm-associated genes in *L. monocytogenes* strains investigated in this study.**

|  |  | ST5 | | | | | | ST37 | | ST204 |
| --- | --- | --- | --- | --- | --- | --- | --- | --- | --- | --- |
| **Gene designation** | **EGD-e homologue** | **4KSM** | **6KSM** | **8KSM** | **10KSM** | **11KSM** | **13KSM** | **1KSM** | **14KSM** | **3KSM** |
| **Cell envelope biosynthesis and homeostasis** | | | | | | | | | | |
| *lmo0644* | *lmo0644* | +^a^ | + | + | + | + | + | + | + | + |
| *lmo2554* | *lmo2554* | + | + | + | + | + | + | + | + | + |
| *lmo2553* | *lmo2553* | + | + | + | + | + | + | + | + | + |
| *dltD* | *lmo0971* | + | + | + | + | + | + | + | + | + |
| *lmo2229* | *lmo2229* | + | + | + | + | + | + | + | + | + |
| *lmo1370* | *lmo1370* | + | + | + | + | + | + | + | + | + |
| *lmo2504* | *lmo2504* | + | + | + | + | + | + | + | + | + |
| *lmo3553* | *lmo3553* | + | + | + | + | + | + | + | + | + |
| **Bacterial quorum sensing (QS) systems** |  |  |  |  |  |  |  |  |  |  |
| *agrD* | *lmo0049* | + | + | + | + | + | + | + | + | + |
| *agrA* | *lmo0051* | + | + | + | + | + | + | + | + | + |
| *degU* | *lmo2515* | + | + | + | + | + | + | + | + | + |
| *luxS* | *lmo1288* | + | + | + | + | + | + | + | + | + |
| **Flagella&motility** |  |  |  |  |  |  |  |  |  |  |
| *flaA* | *lmo0690* | + | + | + | + | + | + | + | + | + |
| *motB* | *lmo0686* | + | + | + | + | + | + | + | + | + |
| *fliP* | *lmo0676* | + | + | + | + | + | + | + | + | + |
| *fliD* | *lmo0707* | + | + | + | + | + | + | + | + | + |
| **Energy generation and intermediary metabolism** |  |  |  |  |  |  |  |  |  |  |
| *atpB* | *lmo2535* | + | + | + | + | + | + | + | + | + |
| *atpE* | *lmo2534* | + | + | + | + | + | + | + | + | + |
| *atpD* | *lmo2529* | + | + | + | + | + | + | + | + | + |
| *lmo2205* | *lmo2205* | + | + | + | + | + | + | + | + | + |
| *purL* | *lmo1770* | + | + | + | + | + | + | + | + | + |
| **Transcription regulators** |  |  |  |  |  |  |  |  |  |  |
| *lmo0734* | *lmo0734* | -^b^ | - | - | - | - | - | + | + | + |
| *lmo1262* | *lmo1262* | + | + | + | + | + | + | + | + | + |
| **Enzymes** |  |  |  |  |  |  |  |  |  |  |
| *cheA* | *lmo0692* | + | + | + | + | + | + | + | + | + |
| *relA* | *lmo1523* | + | + | + | + | + | + | + | + | + |
| **Cell surface proteins** |  |  |  |  |  |  |  |  |  |  |
| *bapL* | *lmo0435* | **-** | **-** | **-** | **-** | **-** | **-** | **-** | **-** | + |
| **SOS response** |  |  |  |  |  |  |  |  |  |  |
| *recA* | *lmo1398* | + | + | + | + | + | + | + | + | + |
| *yneA* | *lmo1303* | + | + | + | + | + | + | + | + | + |

^a^+, present gene/^b^-, absent gene; cutoff of 60% amino acid identity for present genes according to Kuenne et al., 2013.
